# Influence of Adjuvant Therapy in Cancer Survivors on Endothelial Function and Skeletal Muscle Deoxygenation

**DOI:** 10.1371/journal.pone.0147691

**Published:** 2016-01-25

**Authors:** Austin K. Ederer, Kaylin D. Didier, Landon K. Reiter, Michael Brown, Rachel Hardy, Jacob Caldwell, Christopher D. Black, Rebecca D. Larson, Carl J. Ade

**Affiliations:** Department of Health and Exercise Science, The University of Oklahoma, Norman, OK, United States of America; University of Pecs Medical School, HUNGARY

## Abstract

The cardiotoxic effects of adjuvant cancer treatments (i.e., chemotherapy and radiation treatment) have been well documented, but the effects on peripheral cardiovascular function are still unclear. We hypothesized that cancer survivors i) would have decreased resting endothelial function; and ii) altered muscle deoxygenation response during moderate intensity cycling exercise compared to cancer-free controls. A total of 8 cancer survivors (~70 months post-treatment) and 9 healthy controls completed a brachial artery FMD test, an index of endothelial-dependent dilation, followed by an incremental exercise test up to the ventilatory threshold (VT) on a cycle ergometer during which pulmonary ${\dot{\text{V}}\text{O}}_{\text{2}}$ and changes in near-infrared spectroscopy (NIRS)-derived microvascular tissue oxygenation (TOI), total hemoglobin concentration ([Hb]_total_), and muscle deoxygenation ([HHb] ≈ fractional O_2_ extraction) were measured. There were no significant differences in age, height, weight, and resting blood pressure between cancer survivors and control participants. Brachial artery FMD was similar between groups (P = 0.98). During exercise at the VT, TOI was similar between groups, but [Hb]_total_ and [HHb] were significantly decreased in cancer survivors compared to controls (P < 0.01) The rate of change for TOI ${(\Delta \text{TOI}\Delta /\dot{\text{V}}\text{O}}_{\text{2}}\text{)}$ and [HHb] $(\Delta \left[\text{HHb}\right]/{\Delta \dot{\text{V}}\text{O}}_{\text{2}}\text{)}$ relative to ${\Delta \dot{\text{V}}\text{O}}_{2}$ were decreased in cancer survivors compared to controls (P = 0.02 and P = 0.03 respectively). In cancer survivors, a decreased skeletal muscle microvascular function was observed during moderate intensity cycling exercise. These data suggest that adjuvant cancer therapies have an effect on the integrated relationship between O_2_ extraction, ${\dot{\text{V}}\text{O}}_{2}$ and O_2_ delivery during exercise.

## Introduction

Numerous types of cancer are frequently treated with chemotherapy or a combination of chemotherapy and radiation. While these treatment regimens have contributed in part to increased cancer survival rates [1], their use is associated with both acute and long-term cardiotoxicity, which over time may result in late-occurring cardiac complications (for review see Khouri et al. [2]). Similarly, Mulroney et al. (2012) reviewed the potential vascular injury associated with adjuvant treatment and cancer survivors, but the direct effects upon the peripheral vascular system have yet to be fully understood [3]. Despite the evidence of endothelial function as an underlying cardiovascular disease risk factor, only a few studies have investigated the effects of adjuvant treatment on this parameter of cardiovascular health [4–6]. In survivors of childhood cancer, Chow et al. (2006) and Dengle et al. (2008) observed a decreased brachial artery flow-mediated dilation (FMD), a measurement of endothelial-dependent dilation, compared to healthy controls. However, these findings are not universal as others have reported no difference in FMD in breast cancer patients ≈20 mo post-treatment compared to healthy adjuvant therapy naïve controls [7].

During dynamic exercise muscle metabolism is dependent on the integration of convective and diffusive components of the O_2_ transport pathway [8]. An essential component to this movement of O_2_ from atmospheric air to muscle mitochondria is the peripheral microvasculature, which forms a complex three-dimensional network that supports the regulation of tissue perfusion and O_2_ diffusive transport. The adverse effects of adjuvant cancer treatments on the microcirculation have previously been reported in myocardium as Ammar et al. (2011) demonstrated a significant decrease in capillary number in rats exposed to doxorubicin chemotherapy [9]. Similarly, others have reported decreases in nail fold capillary density in patients treated with sunitinib therapy [10]. If similar changes occur within the skeletal muscle capillaries the effects may cause an attenuated blood-myocyte O_2_ and substrate exchange leading to decreases in muscle O_2_ extraction and exercise capacity [11, 12].

Near-infrared spectroscopy (NIRS) is a non-invasive means to evaluate the redox state of tissue oxygenation at the level of the small vessels, capillaries, and intracellular sites of O_2_ transport and utilization [13]. Specifically changes in NIRS-derived deoxygenated hemoglobin and myoglobin (Δ[HHb]) provides a reliable estimate of fractional tissue O_2_ extraction within the microcirculation in the field of interrogation [14–19]. During progressive increases in exercise workload and oxygen uptake (${\dot{\text{V}}\text{O}}_{2}$), evaluation of the fractional O_2_ extraction within the contracting muscle via NIRS can provide valuable insight into skeletal muscle microvascular function. Since chemotherapy and radiation have a known impact on central cardiovascular function, the primary aims of the present investigation were twofold: to evaluate resting vascular function, as assessed via brachial artery flow-mediated dilation, and evaluate changes in muscle deoxygenation via NIRS during moderate intensity ramp exercise in cancer survivors previously treated with adjuvant therapy. We hypothesized that i) cancer survivors would have a decreased brachial artery flow-mediated dilation (FMD) response; and ii) have an altered muscle deoxygenation response during moderate intensity ramp exercise compared to healthy cancer-free controls.

## Materials and Methods

### Subjects

Eight individuals were recruited to a cancer survivor group (7 women) and 9 individuals to a control group (7 women). All cancer survivor participants were recruited from advertisements in the local community and cancer support groups. Confirmation of cancer diagnosis, cancer type, and treatment were obtained from each participant’s current oncologist or family practitioner. Cancer survivor participants were ≥ 2 years from diagnosis with a treatment history consisting of chemotherapy and/or radiation therapy (Table 1). Participants in the control group were recruited via local advertisements. Individuals showing interest with similar age and history of cardiovascular disease as the cancer survivor group were included in the study. All participants were free from all known cardiopulmonary disease, microvascular/peripheral artery disease, COPD, asthma, lung disease, cystic fibrosis, and diabetes. In addition, participants were free of any major signs or symptoms suggestive of cardiovascular, pulmonary, or metabolic disease. Smokers and individuals with poorly controlled hypertension (systolic > 160 mmHg), or currently taking statins were excluded from the study. Also, individuals who reported anemia or symptoms of anemia (e.g., light headed, dizziness, and fainting) were excluded from participation. The number of participants was determined based upon previous studies evaluating microvascular responses during exercise [17, 19] and assuming a physiologically relevant 10–15% difference between groups for NIRS responses at 80% power and an α of 0.05.

**Table 1:** Cancer Survivor Treatment Characteristics.

ID no.	Age (yrs)	Sex	Months post-treatment	Chemotherapy treatment	Radiation treatment
1	56	F	40	Herceptin+Taxotere+ Carboplatin+Femara	No
2	54	F	36	Taxotere+Cytoxan	No
3	59	M	31	Cytoxan+Adriamycin+ Vincristine+Etoposide Prednisone+Ifosfamide+ Carboplatin	No
4	55	F	187	Cytoxin+Adriamycin +Taxane	Yes
5	55	F	41	Cytoxan+Taxol	Yes
6	54	F	35	Adriamyacin+Cytoxan+Taxotere+Abraxane	Yes
7	44	F	136	Cytoxan+Adryamycin +Taxotere	Yes
8	42	F	48	Taxotere+Adriamycin +Cyclophosphamide	Yes

### Study Procedures

All experimental procedures were performed on a single morning following a 4 hour fast and after refraining from exercise, alcohol, and caffeine for at least 12 hours. All tests were performed in a thermoneutral environment (21–23°C). Prior to experimental testing verbal and written consent were obtained from each participant. All procedures were approved by the Institutional Review Board for Research Involving Human Subjects at the University of Oklahoma Health Sciences Center, which conformed to the Declaration of Helsinki.

### Measurements

#### General Characteristics

Body mass index was calculated from height and body mass. Resting arterial blood pressure was measured in the supine position from the average of three brachial artery pressure recordings following a 5 minute resting period [20] (Omron BP785N, Hoofddorp, Netherlands). Current level of physical activity was assessed with the International Physical Activity Questionnaire as previously described by Craig et al. [21].

#### Brachial Artery Flow Mediated Dilation

Arterial endothelial-dependent vasodilator function was evaluated via brachial artery flow-mediated dilation (FMD) using the guidelines established by Harris et al. [22]. Following a 10 min supine rest period an automated rapid cuff inflator was placed on the right arm just proximal to the elbow (Hokanson, Bellevue, WA). Using non-invasive 2D and Doppler ultrasound equipped with a linear array transducer operating in duplex mode at a frequency of 10M Hz and 4.0 MHz, respectively (Logiq S8, GE Medical Systems, Milwaukee, WI), measurements of brachial artery diameter and mean blood velocity were simultaneously performed. Doppler velocity measurements were performed and corrected for an angle of insonation less than 60°. Baseline measurements were performed for 1 min at which point the pneumatic cuff was then inflated to at least 20 mmHg above the resting systolic blood pressure for 5 minutes. Occlusion was confirmed by the absence of a radial pulse. Following the 5 minute occlusion period the cuff was released (< 1 s) and continuous measurements of brachial artery diameter and blood velocity were performed for 2 minutes.

Baseline and post-occlusion brachial artery diameter was calculated at 15 frames per second and averaged into 3 s bins using a commercially available edge-detection and wall-tracking software package, which minimizes investigator bias [Vascular Research Tools 6, (Medical Imaging Applications, Coraville, Iowa, USA)]. FMD was calculated as the highest absolute (mmΔ) and relative (%Δ) mean average 3-s diameter following cuff release in peak brachial artery diameter from the preceding baseline diameter. The baseline and post-occlusion time-averaged mean velocity (in centimeters per second) values over each 3 s contraction cycle were calculated on the ultrasound system using the manufacturer’s on-screen software. The binned diameter and velocity data were time aligned and used to calculate shear rate [Shear rate (s^-1^) = (4 × mean blood velocity (cm/s) / diameter (cm)]. The stimulus eliciting brachial artery dilation following cuff deflation was calculated as the area under the shear rate curve (AUC_SR_) determined using the trapezoidal rule [22]. To normalize brachial artery dilation to the shear stimulus, the FMD response was divided by the cumulative shear rate (%Δmm∙s^-2^)[23].

#### Incremental Exercise

Upon completion of the FMD test, subjects rested ~15 min followed by a ramp incremental exercise protocol on a cycle ergometer (Lode BV, Groningen, The Netherlands). Following a 1 minute resting baseline, subjects pedaled at 60–80 rpm with progressive increases in power output at a rate of 15W min^-1^ until the subject fully expressed their ventilatory threshold (VT). The obtainment of the VT was visually determined in real time as the time at which ${\dot{\text{V}}\text{CO}}_{2}$ increased out of proportion with respect to ${\dot{\text{V}}\text{O}}_{2}$ and there was an increase in ${\dot{\text{V}}\text{E}/\dot{\text{V}}\text{O}}_{2}$ with no increase in ${\dot{\text{V}}\text{E}/\dot{\text{V}}\text{CO}}_{2}$ [24]. Following the incremental test using the same criteria the ${\dot{\text{V}}\text{O}}_{2}$ and work rate (accounting for a 30 s mean response time) for the VT was determined via two independent investigators. The VT was chosen as a physiologic end-point in the present study as it demarcates the boundary between moderate and heavy exercise intensity domains [25]. In addition, the VT is effort independent and is widely used as a submaximal index of exercise capacity in clinical and research applications and is therefore an appropriate exercise end-point [26]. Due to the absence of a physician, population age, and potential risk of adverse responses incremental tests to maximum effort could not be performed. Throughout the incremental test metabolic and ventilatory data were continuously recorded via a gas exchange measurement system (True One 2400, Parvo Medics, Sandy, UT), which was calibrated before each testing session according to the manufacturer’s instructions. During off-line analysis the 30 s mean average of pulmonary ${\dot{\text{V}}\text{O}}_{2}$ was calculated at 50 W and the 30 s preceding the VT.

Near-infrared spectroscopy (NIRS) (OxiplexTS; ISS. Champaign, IL) was used to measure total muscle microvascular hemoglobin + myoglobin concentration ([Hb]_total_), and individual oxygenated ([HbO_2_]) and deoxygenated ([HHb]) concentrations. The NIRS probe was placed longitudinally on the belly of the right *m*. *vastus lateralis* and secured using a cohesive bandage. Location of the *m*. *vastus lateralis* was confirmed manually with palpation during active knee extension and visually via 2D ultrasound. The depth of the muscle was measured from the 2D ultrasound image and used to correct for adipose tissue thickness [27]. No movement of the probe was observed during the exercise test. During the test NIRS data were stored at 25 Hz and averaged into 1 s bins during off-line analysis.

The NIRS system used in the present study utilized light-emitting diodes operating at two wavelengths (690 and 830 nm) with an optical-fiber based light and detector source with a separation of 2.5–4.0 cm which make up the primary elements of the sensor. This system also dynamically determined and incorporated tissue scattering and absorption coefficients into the NIRS variable calculations, which allowed for absolute concentrations of [Hb]_total_ and [HHb] to be calculated (μM) as opposed to relative values. The assumptions and limitations relevant to this measurement technique have been previously discussed in detail [28]. Briefly, the NIRS-derived [HHb] is reflective of changes in muscle deoxygenation within the small arterioles, venules, and capillaries, and of intracellular myoglobin [13]. Due to similar absorption properties of the NIRS light wavelengths, distinction between the hemoglobin and myoglobin cannot routinely be made [29]. In addition, the influence of skin blood flow and volume on the NIRS signal cannot be ignored [30], but has been shown to contribute minimally to the NIRS signal [31].

NIRS during incremental cycling exercise has previously been used to evaluate the redox state of microvascular hemoglobin and intracellular myoglobin [16–18]. Changes in [Hb]_total_ (Δ[Hb]_total_) throughout the incremental test were taken as an index of changes in total microvascular hemoglobin concentration which can occur due to changes in regional blood volume and/or capillary hematocrit. It is important to note that Δ[Hb]_total_ does not provide a measurement of systemic hemoglobin concentration. Changes in [HHb] (Δ[HHb]) were taken as an estimate of skeletal muscle fractional O_2_ extraction [16–19]. During off-line analysis, muscle tissue oxygenation index (TOI) was calculated as TOI = [HbO_2_]/ ([HbO_2_] + [HHb]) x 100. The Δ[Hb]_total_, Δ[HHb], and ΔTOI were calculated as the difference between the value obtained during the initial 30 seconds of the incremental test and the mean average at VT. These differences were then used to calculate the individual rates of concentration change relative to the change in ${\dot{\text{V}}\text{O}}_{2}$. Due to several subject’s inability to remain completely at rest (i.e., no movement of the leg) prior to the start of exercise a true resting condition could not be measured. Since the incremental exercise test began at an unloaded workload followed by a 15 W min^-1^ ramp, the mean work rate during this time was 7.5 W, which is similar to the unloaded cycling baseline used in previous studies [16].

### Statistical Analysis

Statistical analyses were performed using a commercially available software package (SigmaPlot/SigmaStat12.5, Systat Software, Point Richmond, CA). Group differences were determined by unpaired t-tests. Ventilatory, gas exchange, and NIRS responses to incremental exercise were analyzed by two-way repeated measures ANOVAs (group x time), with time as the repeated factor. To identify significant changes in the within and between groups a post hoc Holm-Sidak test was performed. All group data are expressed as mean ± SE, unless otherwise stated. Statistical significance was declared when P < 0.05. Given the sample size and need to detect the smallest meaningful physiological differences, effect size comparisons were also made via Cohen’s *d* with threshold values for small, moderate, and large effects as 0.2, 0.5, >0.8 respectively [32].

## Results

### General Characteristics

Individual cancer survivor characteristics of age, sex, months since last date of treatment, chemotherapy drugs used, and radiation exposure are presented in Table 1. Table 2 describes the baseline characteristics of each group. There were no significant differences in age, height, weight, and BMI between cancer survivor and control participants. Resting systolic, diastolic, and mean arterial pressures were also not different between groups. In the cancer survivor group 4 (50%) individuals were classified as inactive, 2 (25%) as minimally active, and 2 (25%) as active. The control group was composed of 1 (11%) inactive, 3 (33%) minimally active, and 5 (56%) active individuals. The ${\dot{\text{V}}\text{O}}_{2}$ (CS, 1.10 ± 0.07 l min^-1^ *vs*. Control, 1.21 ± 0.11 l min^-1^; *P* = 0.43) and workload at VT (CS, 78.6 ± 5.0 W *vs*. Control, 95.4 ± 10.3 W; *P* = 0.18) were not different between groups, suggesting a similar level of submaximal aerobic fitness (Table 2).

**Table 2:** Participant characteristics.

	CS (*n* = 8)	Control (*n* = 9)	P Value
Age, yr	52.4 ± 6.0	53.3 ± 4.4	0.71
Body mass, kg	70.8 ± 14.5	69.2 ± 9.7	0.78
Stature, cm	166.8 ± 6.4	170.1 ± 9.6	0.42
BMI,	25.3 ± 4.0	24.0 ± 3.2	0.45
Systolic BP, mmHg	120.9 ± 9.8	126.9 ± 17.8	0.41
Diastolic BP, mmHg	78.0 ± 6.0	81.7 ± 8.3	0.32
VT, l min^-1^	1.10 ± 0.19	1.21 ± 0.33	0.43
VT, ml kg^-1^ min^-1^	16.2 ± 4.6	17.4 ± 3.7	0.55
VT, W	78.6 ± 14.2	95.5 ± 31.1	0.18

Data are presented as means ± SE; n, no. of subjects; BMI, body mass index; VT, ventilatory threshold.

### Brachial Artery Flow Mediated Dilation

**Fig 1:**
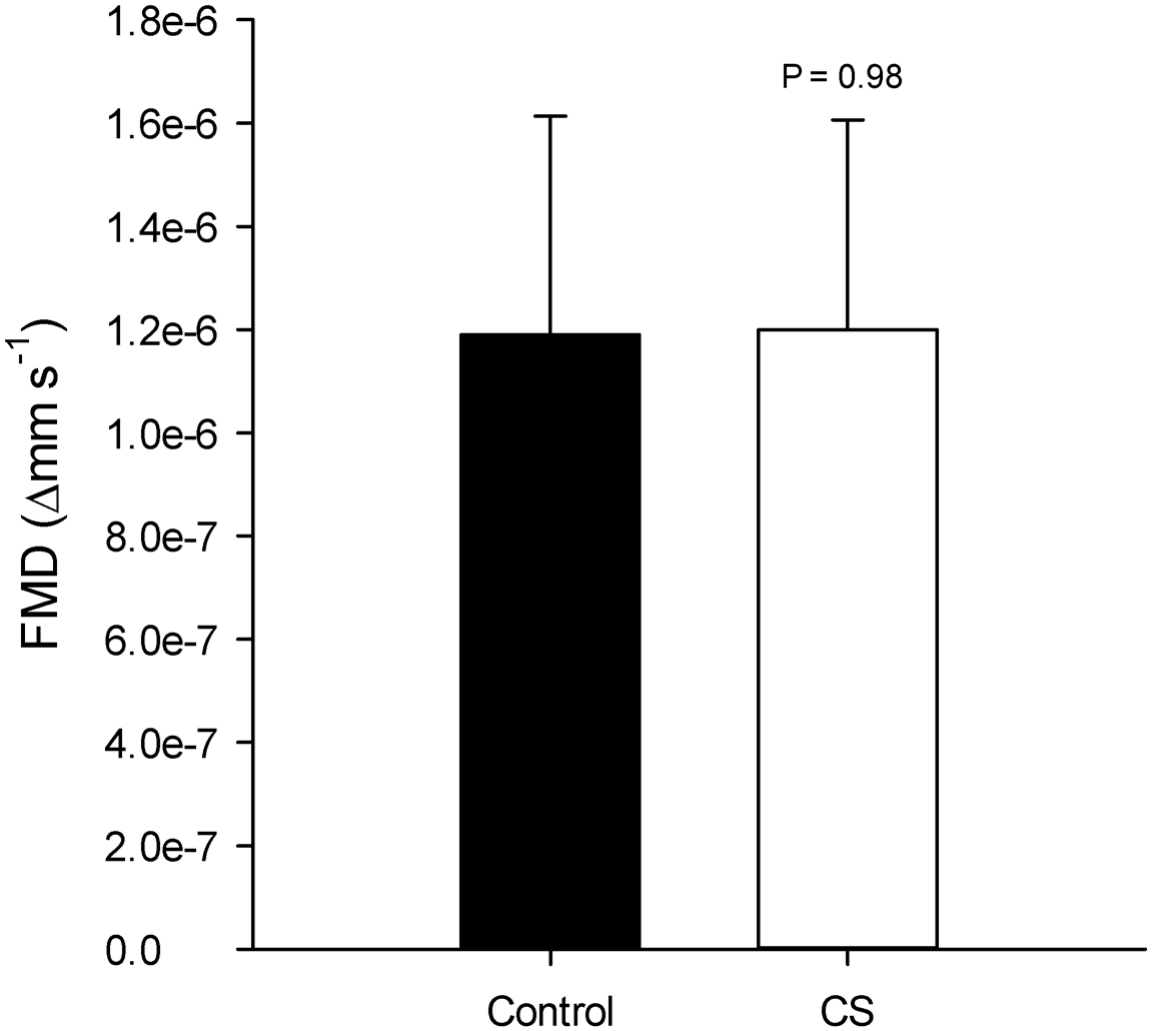
Endothelium-dependent, flow-mediated, brachial artery dilation (FMD) in control and cancer survivors (CS). FMD values are shown normalized for the magnitude of the hyperaemic shear stimulus (i.e. % change in diameter divided by the AUC_SR_).

Brachial artery diameter did not differ between groups prior to cuff inflation (Table 3; *P* = 0.24). Following 5 min of arterial occlusion, the FMD was not different between groups when expressed in absolute (Δmm, Table 3; *P* = 0.29, ES = 0.53) or percentage values (Δ%, Table 3; *P* = 0.21, ES = 0.65). The mean group values of FMD normalized to the shear rate stimulus are illustrated in Fig 1. Similar to the absolute FMD (mm) response, the FMD normalized to the shear rate stimulus (FMD%) was not different between groups (Table 3; *P* = 0.98, ES = 0.01).

**Table 3:** Brachial FMD Responses.

	CS (*n* = 8)	Control (*n* = 9)	*P* Value
Baseline D, mm	3.28 ± 0.15	3.64 ± 0.24	0.24
FMD, mm	0.33 ± 0.09	0.21 ± 0.08	0.29
FMD, %	10.2 ± 2.48	5.77 ± 2.18	0.20
AUC_SR_, s 10^4^	34.2 ± 3.6	22.6 ± 3.1	0.03

Data are presented as means ± SE; n, no. of subjects; D, diameter; FMD, flow-mediated dilation; AUCSR, area under the shear rate curve.

### Exercise Responses

**Fig 2:**
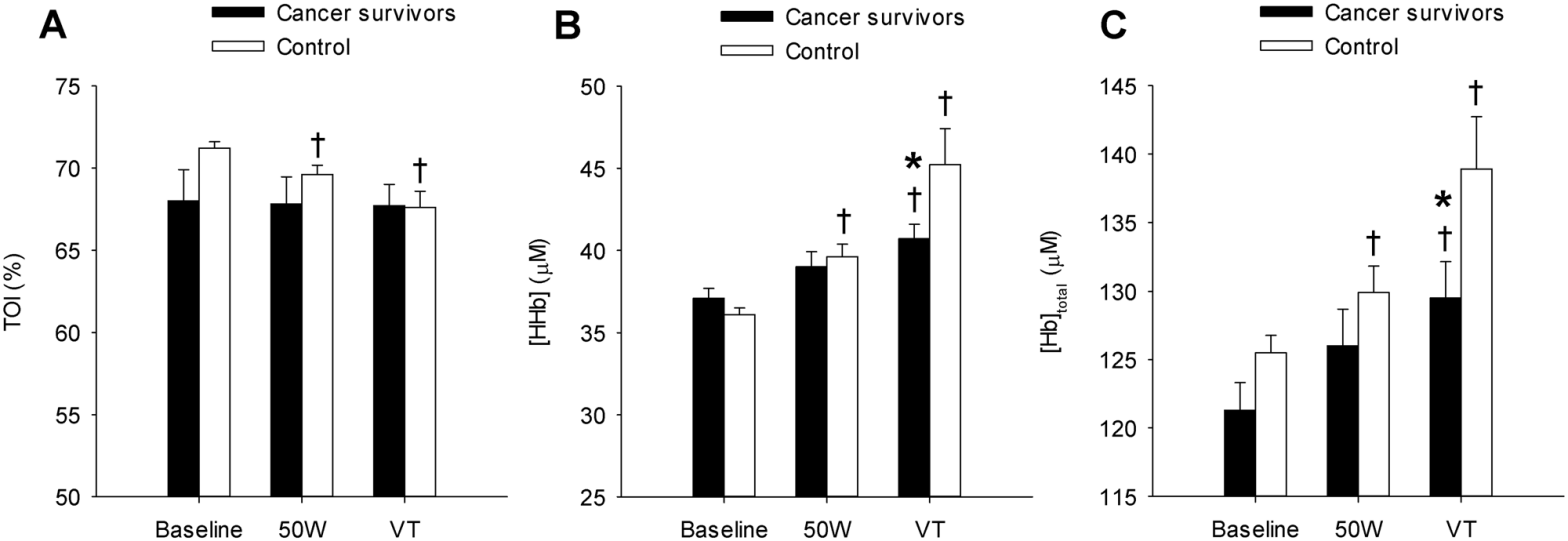
Near-infrared spectroscopy (NIRS)-obtained muscle oxygenation data at baseline, 50 W, and the ventilatory threshold (VT). Tissue oxygenation index (TOI; Panel A) in the cancer survivors remained at baseline levels throughout the test compared to the progressive decrease observed in controls. Deoxygenated hemoglobin ([HHb]; Panel B) was significantly lower in cancer survivors compared to controls at VT. Total hemoglobin ([Hb]_total_; Panel C) at VT was significantly decreased in the cancer survivors compared to controls. Values are mean ± SE; † P<0.05 significantly different compared to baseline. * P<0.05 significantly different compared to controls.

**Fig 3:**
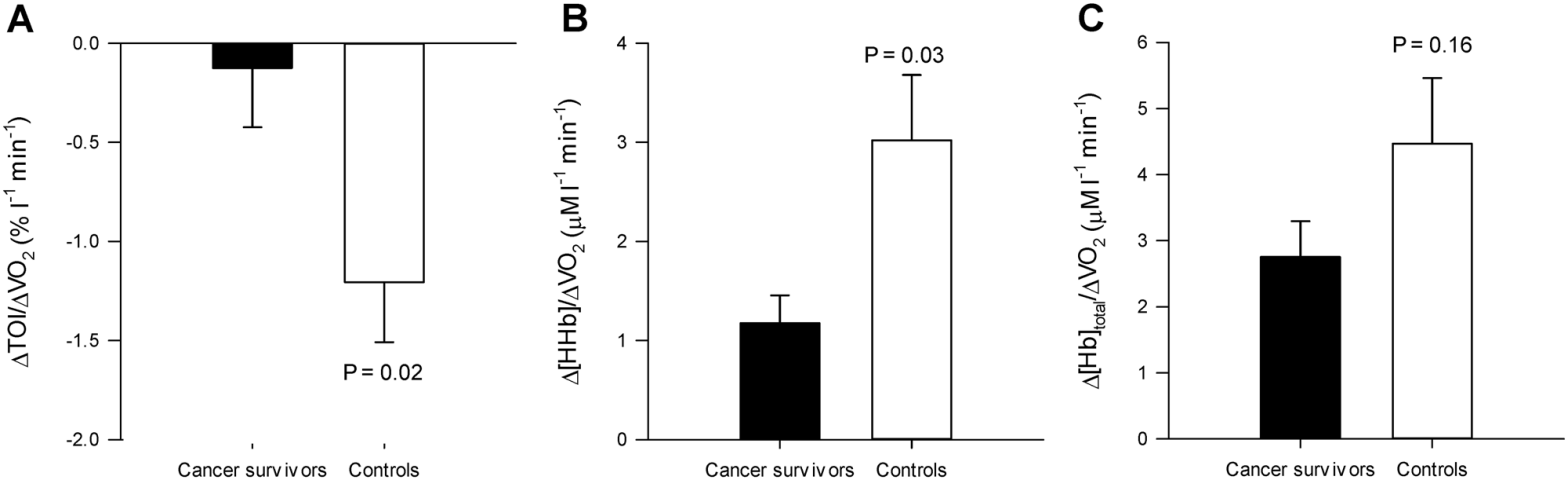
The rate of change for tissue oxygenation index (TOI; Panel A), deoxygenated hemoglobin ([HHb]; Panel B), and total hemoglobin ([Hb]_total_; Panel C) as a function of oxygen uptake (${\dot{\text{V}}\text{O}}_{2}$). Note that the absolute change in TOI and [HHb] as a function of the increase ${\dot{\text{V}}\text{O}}_{2}$ during exercise were significantly less in cancer survivors compared to controls. Values are mean ± SE.

Mean values of NIRS-obtained skeletal muscle microvascular TOI, [HHb], and [Hb]_total_ during the ramp cycling exercise test are illustrated in Fig 2. In the cancer survivors, TOI remained at baseline levels throughout the test compared to the significant decrease observed in controls. There was a significant group-by-work rate interaction for [HHb] (P = 0.01). The muscle [HHb] response, which was used to evaluate changes in fractional O_2_ extraction, was significantly lower in cancer survivors compared to controls at VT (40.6 ± 0.8 vs. 45.2 ± 2.2 μM; *P* = 0.009, ES = 0.98). In the control group, [HHb] was significantly increased above baseline at 50W 36.1 ± 0.4 *vs*. 39.6 ± 0.8 μM; *P* < 0.001) and VT (45.2 ± 2.2 μM; *P* < 0.001). However, [HHb] was only significantly increased above baseline at the VT in the cancer survivors (37.1 ± 0.6 *vs*. 40.6 ± 0.9 μM; *P* = 0.04) suggesting a delayed increases in fractional O_2_ extraction in the cancer survivor group compared to controls. Muscle [Hb]_total_ significantly increased at 50W (125.5 ± 1.3 *vs*. 129.9 ± 1.9 μM; *P* = 0.04) and VT (138.9 ± 3.8 μM; *P* < 0.001) relative to baseline in controls, whereas [Hb]_total_ only increased at VT in the cancer survivors (121.3 ± 2.0 *vs*. 129.5 ± 2.7; *P* = 0.002). The [Hb]_total_ at VT was significantly decreased in the cancer survivors compared to controls (129.5 ± 2.7 *vs*. 138.9 ± 3.8 μM; *P* = 0.02, ES = 0.96). The rate at which TOI (CS, -0.13 ± 0.3 *vs*. Control, -1.21 ± 0.3 μM l^-1^ min^-1^; *P* = 0.02, ES = 1.19) and [HHb] (CS, 2.76 ± 0.54 *vs*. Control, 4.47 ± 0.99 μM l^-1^ min^-1^; *P* = 0.03, ES = 1.22) increased for a given increase in ${\dot{\text{V}}\text{O}}_{2}$ throughout the ramp exercise test were significantly less in cancer survivors compared to controls (Fig 3).

## Discussion

The purpose of this study was to evaluate endothelial function and the skeletal muscle deoxygenation responses to moderate intensity ramp exercise in a group of cancer survivors and healthy controls. This study has three major findings. First, brachial artery FMD, a non-invasive measurement of endothelial function, was not different between cancer survivors and healthy controls. This finding does not support the first hypothesis. The second key finding was that skeletal muscle microvascular [Hb]_total_ and [HHb] were significantly decreased during dynamic cycle ergometry in the cancer survivors compared to healthy controls. These decreases suggest that muscle microvascular hemoglobin concentration and muscle O_2_ extraction were lower in the cancer survivor group. Lastly, these differences resulted in decreased ΔTOI and Δ[HHb] relative to ${\Delta \dot{\text{V}}\text{O}}_{2}$ in the cancer survivor group. Taken together these findings suggest that the muscle microvascular responses to dynamic exercise are attenuated in the cancer survivors compared to healthy controls. In total these conclusions are consistent with the second hypothesis that cancer survivors have an altered muscle deoxygenation response to moderate intensity exercise. The decreased muscle deoxygenation suggests the presence of some alteration in the balance between O_2_ delivery, O_2_ extraction, and O_2_ utilization during exercise in cancer survivors, which to our knowledge, represents the first evidence of potential muscle microvascular toxicity in cancer survivors treated with adjuvant treatments.

### Endothelial function

Treatment with certain chemotherapies can elicit endothelial damage and vascular dysfunction. Ito et al. [33] and Gibson et al. [34] exposed rat aortas to anthracycline chemotherapy, specifically doxorubicin, and demonstrated significant decreases in acetylcholine-induced endothelial-dependent relaxation within hours of injection. Similarly, Duquaine et al. [5] demonstrated a significantly decreased brachial artery FMD immediately following infusion of Adriamycin chemotherapy in breast cancer patients. Like chemotherapy, radiation has also been shown to acutely decrease endothelium-dependent vascular function [35, 36].

Similar to the findings that chemotherapy can acutely impact vascular function, Chow et al. [4] observed a decreased brachial artery FMD responses several months following anthracycline chemotherapy in pediatric cancer patients. While suggestive of long-term adverse vascular effects of chemotherapy, their study can only be generalized to individuals treated prior to 21 yrs old. In the present study, brachial artery FMD was unaffected by prior exposure to chemotherapy or a combination of chemotherapy and radiation compared to controls. In a similar study design, Jones et al [7] demonstrated no difference in brachial artery FMD in breast cancer patients approximately 20 mo following chemotherapy compared to healthy controls. The present findings in combination with those of Jones et al. [7] suggest that the long-term effects of chemotherapy and radiation in the major conduit arteries may be minimal, which may indicate that the vasculature, at least within the brachial artery, may have the ability to restore its dilative qualities in the years following treatment. One experimental consideration to note when interpreting the similar brachial artery FMD between cancer survivors and controls is the vascular heterogeneity that exists between limbs. While regional brachial artery FMD traditionally has been used as an index of global vascular health, recent evidence strongly cautions against extrapolating the findings from a single limb to the whole body since it is now recognized that endothelial and vascular smooth muscle exhibit heterogeneity across the peripheral vasculature[37]. Specifically, Newcomer et al. (2004) demonstrated that the endothelium-dependent vascular responses in the femoral artery are significantly different compared to the brachial artery[37]. It is therefore critical that the findings from the present investigation are not used as a comprehensive index of global vascular health and that a more compressive evaluation of vascular function in multiple vascular beds is required in the cancer survivor population.

### Microvascular responses to exercise

The VT expressed as both a work rate and ${\dot{\text{V}}\text{O}}_{2}$ were similar between groups. The VT provides a non-invasive measurement of the lactate threshold, which together demarcate the boundary between moderate and heavy-intensity exercise domains and occurs at ~ 50% ${\dot{\text{V}}\text{O}}_{\text{2}}\text{max}$ [24]. In addition, the VT is effort independent and is widely used as a submaximal index of exercise capacity in clinical and research applications [26]. The similar VT between groups is in line with the similar levels of self-reported physical activity in each group.

#### NIRS

During incremental exercise muscle [HHb] progressively increases linearly as a function of exercise intensity up to ~75–90% ${\dot{\text{V}}\text{O}}_{\text{2}}\text{max}$ and reflects skeletal muscle microvascular O_2_ extraction [16, 17]. Similar to previous work, the present study observed significant increases in [HHb] throughout the incremental exercise test up to the VT. However, at the VT muscle [HHb] was significantly lower in the cancer survivors compared to healthy controls, suggesting a significantly decreased skeletal muscle microvascular O_2_ extraction at the higher exercise intensity (Fig 2). This conclusion is supported by the decreased Δ[HHb] for a given increase in ${\Delta \dot{\text{V}}\text{O}}_{2}$ in the cancer survivors compared to controls, which suggests that the rate at which microvascular O_2_ extraction increased in proportion to ${\dot{\text{V}}\text{O}}_{2}$ was significantly attenuated in the cancer survivors. These findings indicate that the ability to increase fractional O_2_ extraction as the metabolic demands of exercise increased may be impaired in this patient population.

The differences in skeletal muscle microvascular [HHb] during exercise observed in the current study are similar to those reported in other patient populations [38] and following extended periods of bedrest [39, 40]. In post-myocardial infarction patients the increase in muscle [HHb], evaluated using NIRS, was significantly lower compared to healthy controls during the transition from rest to peak cycling exercise, which was also significantly correlated with peak aerobic capacity [38]. Similarly, in healthy individuals exposed to bedrest, the change in skeletal muscle microvascular [HHb] responses in the *m*. *vastus lateralis*, also evaluated using NIRS, during dynamic exercise were significantly decreased compared to pre bed rest values. Taken together, these previous investigations highlight the adverse microvascular changes associated with acute cardiovascular insult and physical inactivity, both of which can occur following adjuvant cancer treatment [41]. The findings of the present study, which observed a significantly decreased Δ[HHb] as a function of ${\Delta \dot{\text{V}}\text{O}}_{2}$ in a group of cancer survivors compared to untreated cancer treatment naïve controls provides evidence that factors associated cancer survival and prior treatment with adjuvant therapy adversely impact the factors associated with O_2_ extraction. Given that the profile of microvascular O_2_ extraction during incremental exercise is impart dependent upon muscle ${\dot{\text{V}}\text{O}}_{2}$ and muscle blood flow; these findings suggest that the decreased O_2_ extraction in the cancer survivors during moderate intensity exercise may have been compensated by an increased muscle blood flow. While muscle blood flow was not measured in the present investigation, the similar FMD responses between the cancer survivors and controls suggests that endothelium-dependent vasodilation during exercise may have also been similar. Also during moderate intensity exercise a substantial cardiac output reserve exists, which may have allowed for a compensatory increased muscle blood flow in response to the decreased O_2_ extraction for a given metabolic rate in the cancer survivors, thus allowing ${\dot{\text{V}}\text{O}}_{2}$ to appropriately increase with increases in workload. Future investigations will need to determine if the control of blood flow is altered during dynamic exercise and how it impacts the muscle [HHb] response in cancer survivors.

#### Determinants of O_2_ extraction

During dynamic exercise O_2_ extraction is dependent on the integration of ‘central’ and ‘peripheral’ factors which include: muscle DO_2_, capillary muscle O_2_ conductance, muscle blood flow, blood flow heterogeneity, arterial O_2_ content, and muscle oxidative capacity and is can be mathematically expressed as: 1 –e−^DO2/(β∙Q)^ (DO_2_, O_2_ diffusing capacity; $\dot{Q}$, blood flow; β, slope of the O_2_ dissociation curve) [42–44]. In the present study’s group of cancer survivors, it is plausible that changes in both ‘central’ and ‘peripheral’ factors may have contributed to the decreased [HHb]. A key factor in O_2_ extraction is O_2_ diffusing capacity (DO_2_), which is thought to be primarily determined by microvascular hematocrit (Hct) [11]. At rest, microvascular Hct is less than systemic Hct, which increases with muscular contraction and plays a key role in the contraction-induced increases in muscle DO_2_ [12]. Previous investigations have highlighted that changes in NIRS measured [Hb]_total_ reflect increases in microvascular Hct given that muscle myoglobin concentration presumably does not change with exercise [29]. In the present study [Hb]_total_ was decreased at the VT in the cancer survivors compared to healthy controls, which is suggestive of a decreased microvascular hemoglobin concentration and subsequent capillary hematocrit. Therefore, the decreased fractional O_2_ extraction observed during moderate intensity exercise in cancer survivors may be due in part to decreases in muscle DO_2_ owing to a decreased microvascular Hct. In addition to potential decreases in microvascular Hct, the decreased [HHb] response during exercise in the cancer survivors could be due to additional factors including heterogeneity in microvascular mean transit time, blood flow, blood flow distribution, capillary density, and decreased mitochondrial activity [45–47]. The measurement depth of the NIRS technique is limited to approximately half the distance between the light source and detector, which with the present study’s NIRS system is equal to 2 cm. Any heterogeneity in mean transit time or blood flow would subsequently result in regional differences in the [HHb] response and suggest that the decreased [HHb] observed during exercise in our group of cancer survivors compared to healthy controls may only exist in the superficial portions of the *m*. *vastus lateralis*.

The integrated relationship between the profiles for ${\dot{\text{V}}\text{O}}_{2}$, blood flow, and muscle [HHb] during incremental exercise dictate that the lower $(\Delta \left[\text{HHb}\right]/{\dot{\text{V}}\text{O}}_{\text{2}}\text{)}$ in the cancer survivors was likely compensated by a greater increase in muscle blood flow for a given increase in ${\dot{\text{V}}\text{O}}_{2}$. Thus, it is unlikely that the decreased muscle [HHb] relative to ${\dot{\text{V}}\text{O}}_{2}$ would significantly limit exercise during moderate intensity exercise. This is supported by the finding that our submaximal index of exercise capacity (i.e., VT) was similar between groups. However, had exercise continued to near maximal exercise intensities, when the ability to increase muscle blood flow may become compromised, the altered microvascular function may have limited peak exercise capacity in a similar manner as that reported in post-myocardial infarction [38].

### Experimental considerations

The strengths of the study include the similar levels of self-reported physical activity and measured VTs in a diverse group cancer survivors and controls. This is important given that fitness level alone can impact measurements of brachial artery FMD [48] and NIRS derived measurements of skeletal muscle deoxygenation [17]. However, there are several important limitations to this study. First, the type of treatment, while documented, was not controlled for, nor was the type of cancer. This was done so that the results of this study would be more relatable to the general cancer-survivor population. Second, the population measured was predominantly female and is therefore not representative of cancer survivors as a whole [49] and the sample size was also modest, but very similar to previous investigations evaluating the NIRS response to dynamic exercise and is supported by our a prior sample size calculations [16, 18, 19, 50]. Third, much of the previous research involving NIRS measurements of skeletal muscle microvascular function utilized a ramp cycling protocol to ${\dot{\text{V}}\text{O}}_{\text{2}}\text{max}$, which is a historically relevant measurement of integrative cardiovascular function. The present study was limited to submaximal exercise intensities and it is therefore unknown as to how the results may have differed during a maximal incremental test. Fourth, the NIRS analysis and interpretation is associated with certain methodical assumptions and limitations [28, 51]. Briefly, the NIRS-derived [HHb] is reflective of changes in hemoglobin oxygenation within the small arterioles, venoules, capillaries, and intracellular myoglobin due to similar absorption properties of the NIRS light wavelengths, thus preventing distinction between the two [29]. Also, the NIRS probe was attached to the mid portion of the right *m*. *vastus lateralis* and only allowed for a portion of the exercising muscle to be investigated. It must be recognized that spacial heterogeneities exist and that the observed differences between cancer survivors and control groups could be due to differences in O_2_ transport, diffusion, and utilization across different locations within the muscle [46]. Lastly, blood flow was not measured. Since changes in muscle [HHb] may be the result of several factors, including alterations in blood flow, it this measurement would have provided valuable insight into the underlying mechanisms altering the microvascular responses during exercise in the cancer survivors. However, this measurement, while important, could not be performed in the present study due to the invasiveness and technical issues associated with the determination of exercising blood flow during dynamic cycling exercise.

### Summary

From the present study in can be concluded that the pattern of microvascular redox status, evaluated via NIRS, during a moderate intensity ramp exercise test is influenced by prior exposure to adjuvant cancer therapy. In cancer survivors skeletal muscle microvascular [Hb]_total_ and [HHb] were compared to healthy controls. In addition, cancer survivors demonstrated decreased rates of change for TOI and [HHb] relative to metabolic rate, probably due alterations in microvascular function, fiber type distribution, intracellular oxidative capacity, or muscle blood flow. Since skeletal muscle [HHb] is an estimate of O_2_ extraction and since ${\dot{\text{V}}\text{O}}_{\text{2}}$, blood flow, and O_2_ extraction are related, our data suggest that adjuvant cancer therapies has an effect on the integrated relationships required for the maintenance of dynamic exercise. The mechanistic reasons for these findings and the functional consequences can only be speculated at this point and future investigations should focus providing further mechanistic insight into the long-term effects of adjuvant cancer therapies.

## Supporting Information

S1 TREND Checklist TREND statement Checklist. (PDF)

S1 Table Results of the control and CS groups. Data are presented as means ± SE and ± SD; n, no. of subjects; BMI, body mass index; VT, ventilatory threshold; D, diameter; FMD, flow-mediated dilation; AUCSR, area under the shear rate curve; [THC], total hemoglobin+myoglobin; [HHb], deoxygenated hemoglobin+myoglobin; TOI, tissue oxygenation index.
(XLSX)
